# Novel HLA-B7-restricted human metapneumovirus epitopes enhance viral clearance in mice and are recognized by human CD8^+^ T cells

**DOI:** 10.1038/s41598-021-00023-0

**Published:** 2021-10-21

**Authors:** Margot Miranda-Katz, John J. Erickson, Jie Lan, Alwyn Ecker, Yu Zhang, Sebastian Joyce, John V. Williams

**Affiliations:** 1 Department of Pediatrics, University of Pittsburgh School of Medicine, UPMC Children’s Hospital of Pittsburgh, 4401 Penn Ave, Rangos 9122, Pittsburgh, PA 15224 USA; 2 Department of Pathology, Microbiology and Immunology, Vanderbilt University School of Medicine, Nashville, USA; 3 Department of Veterans Affairs, Tennessee Valley Healthcare System, Nashville, USA; 4 Vanderbilt Institute for Infection, Immunity, and Inflammation (VI4), Nashville, TN 37232 USA; 5 Institute for Infection, Inflammation, and Immunity in Children (i4Kids), Pittsburgh, PA 15224 USA

**Keywords:** Viral host response, MHC class I, Cytotoxic T cells, Viral infection

## Abstract

Human metapneumovirus (HMPV) is a leading cause of acute lower respiratory tract illness in children and adults. Repeated infections are common and can be severe in young, elderly, and immunocompromised persons due to short-lived protective humoral immunity. In turn, few protective T cell epitopes have been identified in humans. Thus, we infected transgenic mice expressing the common human HLA MHC-I allele B*07:02 (HLA-B7) with HMPV and screened a robust library of overlapping and computationally predicted HLA-B7 binding peptides. Six HLA-B7-restricted CD8^+^ T cell epitopes were identified using ELISPOT screening in the F, M, and N proteins, with M_195–203_ (M195) eliciting the strongest responses. MHC-tetramer flow cytometric staining confirmed HLA-B7 epitope-specific CD8^+^ T cells migrated to lungs and spleen of HMPV-immune mice. Immunization with pooled HLA-B7-restricted peptides reduced viral titer and protected mice from virulent infection. Finally, we confirmed that CD8^+^ T cells from HLA-B7 positive humans also recognize the identified epitopes. These results enable identification of HMPV-specific CD8^+^ T cells in humans and help to inform future HMPV vaccine design.

## Introduction

Human metapneumovirus (HMPV) is a pneumovirus discovered in 2001 and a major cause of acute respiratory infection^1^. Young children, elderly, and immune compromised individuals are at high risk for severe HMPV disease^2–10^. All individuals have been exposed to HMPV by the age of 5 years^1,11^, but humoral immunity does not fully protect against reinfection of adults^12–14^. The CD8^+^ T cell response contributes to HMPV clearance^15,16^ and persons with diminished T cell immunity, such as transplant recipients and cancer patients, can experience severe and fatal HMPV infection^5,6,8,17^. Programmed Cell Death-1 (PD-1) and other inhibitory receptors mediate impairment of lung CD8^+^ T cells following HMPV infection in mice and humans^18,19^, providing further evidence of the importance of T cells in viral clearance.

Live-attenuated HMPV vaccines have been tested in clinical trials, but no licensed vaccines or therapeutics exist. Recombinant HMPV fusion (F) protein vaccines induce neutralizing antibodies^20,21^, but their ability to elicit protective CTLs in humans is unclear. T cell responses are thought to be important for protection. CTL epitope vaccines reduced viral titers in mice^22^, and virus-like particles (VLP) generated both antibody and T cell responses against HMPV^23,24^. To track and study CD8^+^ T cell responses to vaccines and to natural HMPV infection in humans, it is necessary to map MHC I-restricted viral epitopes. Only a few MHC-I restricted HMPV epitopes have been identified in humans^25–27^.

In this study, we used transgenic mice expressing human HLA-B*07:02 and lacking the classical mouse MHC class I molecules H-2 K^b^ and D^b^ (Kb^−/−^/Db^−/−^), to identify novel CD8^+^ T cell epitopes recognized during HMPV infection. Immunization of mice with the epitopes reduced lung viral titers following challenge. We found that human CD8^+^ T cells of HLA-B*07:02 positive subjects recognized the epitopes by ELISpot and tetramer staining. Our results show that the transgenic mouse is a useful model for identification of HLA-B*07:02-restricted HMPV epitopes and suggest novel targets for vaccination against HMPV.

## Materials and methods

### Mice, infections, and cells

HLA-B*07:02 transgenic mice on a C57BL/6 (B6) background were obtained from Institut Pasteur^28^. All animals were bred and maintained in specific pathogen-free conditions under guidelines approved by the AAALAC and the University of Pittsburgh Institutional Animal Care and Use Committee. Six to 12-week-old age- and gender-matched animals were used in all experiments. HMPV (strain TN/94-49, genotype A2) was grown and titered in LLC-MK2 cells as previously described^29^. For all experiments, mice were anesthetized with intraperitoneal ketamine-xylazine or inhaled isoflurane and infected intranasally (i.n.) or intratracheally (i.t.) with 1 × 10^6^ PFU of HMPV. Viral titers in infected mouse lungs were measured by plaque titration as described previously^29^. HLA-characterized PBMCs from unique human donors were obtained from Cellular Technology Limited (C.T.L.). All experimental protocols were approved by the Vanderbilt University IACUC or the University of Pittsburgh IACUC. The study was carried out in compliance with the ARRIVE guidelines.

### Epitope prediction and peptides

The online prediction algorithms SYFPEITHI (http://syfpeithi.de)^30^, BIMAS (http://www.bimas.cit.nih.gov)^31^ and IEDB (using the ANN, ARB, and SMM algorithms)^32^ were used to generate HMPV epitope predictions for the HLA-B*0702^33^, H2-D^b^ and H2-K^b^ alleles using the amino acid sequences for the nucleocapsid (N), phosphoprotein (P), matrix (M), F, matrix 2 (M2-1, M2-2), short hydrophobic (SH), glycoprotein (G), and polymerase (L) open reading frames from the HMPV A2 strain NL/00/17 (GenBank accession number FJ168779). The top 148 HLA-B*0702-restricted 8-10 amino acid long predictopes were synthesized (by Mimotopes or Genscript) to > 90% purity and tested for in vitro MCH-I binding by iTopia (Coulter)^33^. Of these, 78 exhibited in vitro binding (Supplemental Table 1) and were further tested along with two irrelevant RSV peptides using IFNγ ELISpot.

### IFNγ ELISpot

ELISpot assays were performed as previously described^18^. The mitogen concanavalin A (ConA, Sigma) was used as a positive control, while stimulation with irrelevant RSV peptides served as a negative control. Peptides were added at a final concentration of 10 µM. The average number of spots counted from the negative control wells was subtracted from each of the HMPV epitope wells, and the data were expressed as spot-forming cells (SFC) per 10^6^ lymphocytes. The antibodies used for the murine ELISpot were anti-interferon-γ (IFN-γ) monoclonal antibody (mAb) clone AN-18 (5 mg/ml; eBioscience) and biotinylated anti-IFN-γ mAb R4-6A2 (2 mg/ml; eBioscience). For the human ELISpot, the antibodies were anti-IFN-γ mAb 1-D1K (5 mg/ml; Mabtech) and biotinylated anti-IFN-g mAb 7-B6-1 (2 mg/ml; Mabtech).

### Flow cytometry

Cells were isolated from lungs and spleens of infected animals as previously described^18^. Briefly, lungs were rinsed in R10 medium (RPMI-1640 plus 10% FBS, 2 mM glutamine, 50 mg/ml gentamicin, 2.5 mg/ml amphotericin B, and 50 mM β-mercaptoethanol (Life Technologies), minced with a scalpel, and incubated with 2 mg/ml collagenase A (Roche) and 20 mg/ml DNase (Roche) for 1 h at 37 °C. Single-cell suspensions of spleens and digested lungs were obtained by pressing through a steel screen (80 mesh) and then passing over a 70-μm nylon cell strainer. Erythrocytes were lysed using Red Blood Cell Lysis Buffer (Sigma-Aldrich). For labeling of HMPV-specific CD8^+^ T cells, single cell suspensions of mouse lung or spleen were stained with violet LIVE/DEAD dye (Life Technologies), Fc blocked with 1 mg per 10^6^ cells anti-CD16/32 (BD Biosciences) and incubated with APC-labeled HLA-B*0702 tetramers (0.1–1 mg/ml), anti-CD8a (clone 53–6.7, BD Biosciences), anti-CD3 (clone 145-2C1, BD Biosciences) and anti-CD19 (clone 1D3, eBioscience)^18^. Surface/tetramer staining was performed for 1 h at room temperature in PBS containing 2% FBS and dasatinib at 50 nM^34^. Staining for HMPV-specific CD8^+^ T cells was normalized to the binding of an irrelevant APC-labeled vaccinia virus (VV) tetramer. For all cell populations, FSC and SSC gating were used to define cells of appropriate size and shape. All flow cytometric data were collected using an LSRII or Fortessa (BD Biosciences) and analyzed with FlowJo software (Tree Star).

### Mouse immunization

Bone marrow derived DCs were generated as previously described^18^. BMDCs were incubated overnight with 500 ng/ml LPS (Sigma-Aldrich) and 10 μM peptide to mature and load the DCs with individual epitope peptides from vaccinia virus or HMPV. B7tg mice were immunized subcutaneously with 1 × 10^6^ peptide-loaded, LPS-matured DCs each and challenged with HMPV 14 days later. For peptide vaccination, mice were injected i.p. with different doses of pooled peptides, 10 µg LPS, and PBS in a total volume of 100 µl and allowed to rest for 4 weeks before challenge with HMPV. On day 5 post-challenge, mice were euthanized, and lungs harvested for virus titration.

### Tetramer enrichment

Human HLA-typed PBMCs were obtained from a commercial source (Cellular Technology Limited), thawed, washed with RPMI, re-suspended in 200 ml FACS (PBS containing 2% FBS and 50 nM dasatinib), and stained 30 min at RT with 10 ml of APC-labeled tetramer. Cells were then subjected to APC-tetramer enrichment using EasySep Human APC Positive Selection Kit (Stemcell Technologies). Both unenriched and enriched fractions were collected for flow cytometric analysis as described above.

### Statistical analyses

Data analysis was performed using Prism v9 (GraphPad Software). Groups were compared using unpaired t test or one-way ANOVA with post-hoc Tukey test for multiple comparisons. P < 0.05 was considered significant by convention.

## Results

### HMPV peptides are recognized by IFNγ-producing cells in HLA-B7tg mice

**Figure 1:**
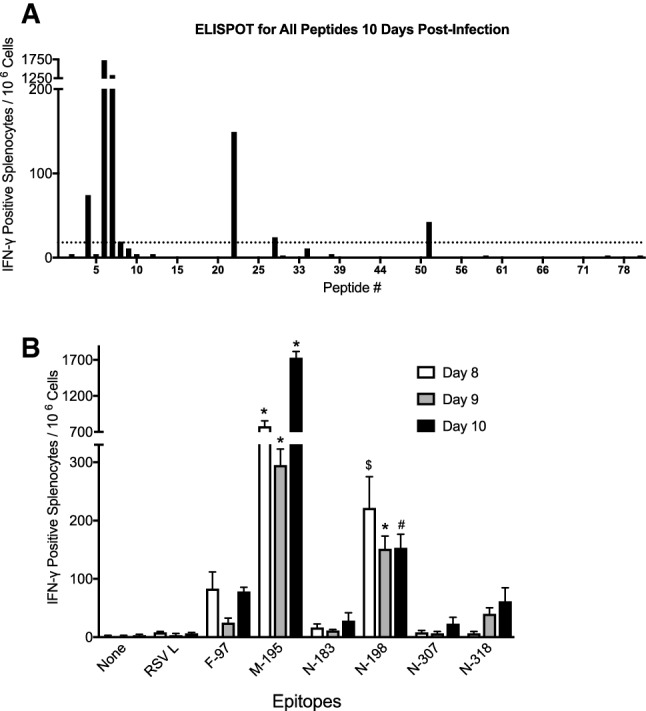
HMPV peptides are recognized by IFNγ-producing cells in HLA-B7 transgenic mice. B6-Kb^0^Db^0^;HLA-B*07:02 transgenic (B7tg) mice were infected i.n. with 1 × 10^6^ PFU HMPV and splenocytes isolated 10 days post-infection. HMPV immune cells were screened for IFNγ release via ELISPOT assay using (**A**) 80 HLA-B*07:02 predictopes or (**B**) 6 selected HMPV epitopes and an irrelevant RSV epitope. Results indicate spot forming cells (SFC) per 10^6^ splenocytes following stimulation with the indicated peptide. Data are combined from 3 independent experiments with 5 pooled mouse spleens per experiment. *p < 0.0001, ^$^p < 0.001, ^#^p < 0.05 compared to none.

HLA-B7tg mice were infected with HMPV via the respiratory tract and splenocytes collected on day 10 were plated with predictope peptides in triplicate in an IFNγ ELISPOT assay. Six peptides generated a response significantly above background (Fig. 1; Table 1), with a peptide from the matrix (M) protein M_195-203_ (M195) demonstrating the highest response. Testing of splenocytes collected on day 8, 9, or 10 post-infection confirmed that stimulation with M195 and N_198-206_ (N198) peptides produced a significantly increased number of HMPV-immune splenocytes releasing IFNγ (Fig. 1B). F97, N183, N307, and N318 peptides elicited an increased number of spots above background that did not reach statistical significance.

**Table 1:** HMPV peptides recognized by IFNγ-producing cells in HLA-B*07:02 mice.

Name	Protein	Position	Sequence
M195	M	195–203	APYAGLIMI
F97	F	97–105	NPRQSRFVL
N183	N	183–191	TVRRANRVL
N198		198–206	YPRMDIPKI
N307		307–315	SPKAGLLSL
N318		318–326	CPNFASVVL

### Fluorescently labeled pB7.2 tetramers identified CD8^+^ T cells in B7tg mice

**Figure 2:**
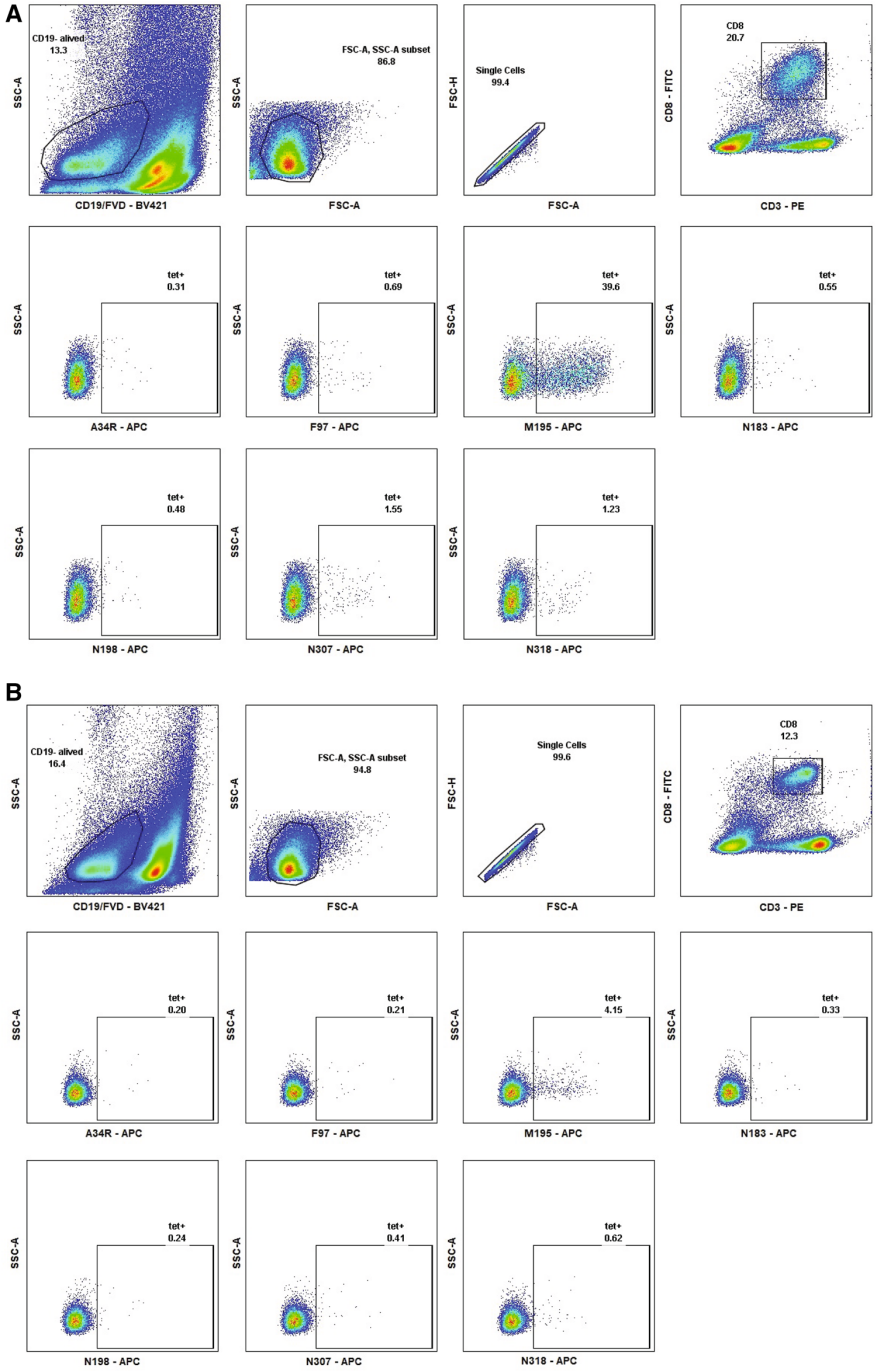
HMPV-specific T_CD8_ identified with fluorescently labeled HLA tetramer molecules in B7tg mice. B7tg mice were infected i.n. with 1 × 10^6^ PFU HMPV and lungs (**A**) and spleens (**B**) collected on day 10 post-infection. Live/dead dye and fluorescently labeled CD8 and CD3 antibodies were used to identify live CD8^+^ T lymphocytes and fluorescently labeled HMPV-specific tetramers, as well as an irrelevant vaccinia virus (A34R) tetramer, were used to identify HMPV epitope-specific T_CD8_.

**Figure 3:**
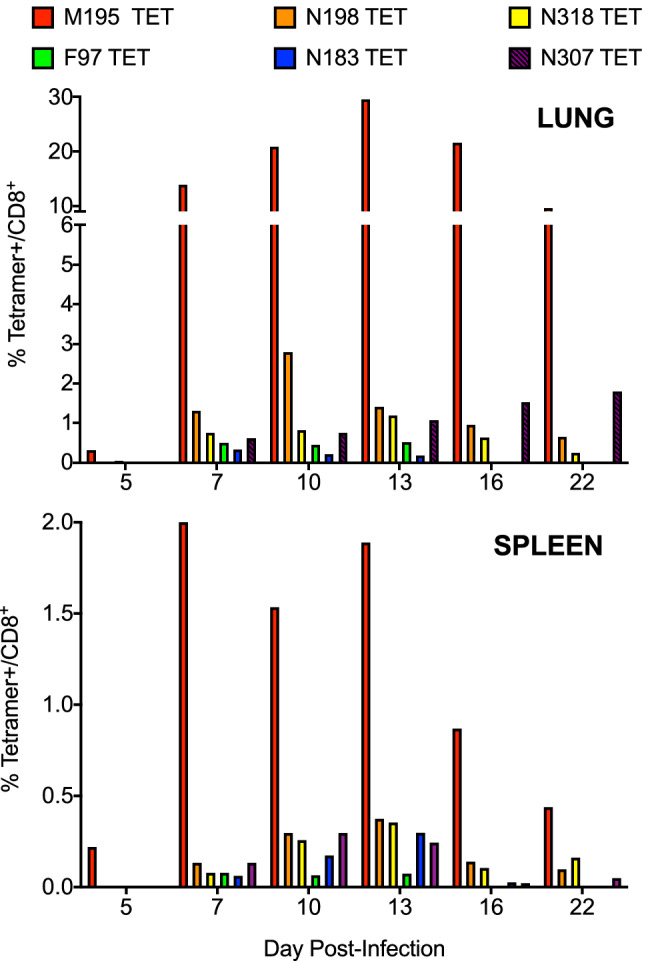
HMPV-specific T_CD8_ peak in the lung on day 13 in B7tg mice. B7tg mice were infected i.n. with 1 × 10^6^ PFU HMPV and lungs and spleens collected on days 5, 7, 10, 13, 16, and 22 post-infection. Live/dead dye and fluorescently labeled CD8 and CD3 antibodies were used to identify live CD8^+^ T lymphocytes and fluorescently labeled HMPV-specific tetramers used to enumerate epitope-specific T_CD8_. Data are representative of two independent experiments with N = 3–5 mice per experiment.

To assess whether HLA B7-restricted CD8^+^ T cells recognize the identified HLA B7-restricted peptides in a direct binding assay, we generated fluorescently labeled tetramers loaded with the antigenic epitopes of interest identified above. B7tg mice were infected with HMPV and their lungs and spleens were harvested on day 10 post-infection. Live cells CD19^−^ cells within the lymphocyte gate by forward/side scatter were gated on CD3^+^, CD8^+^, and tetramers loaded with an irrelevant vaccinia virus peptide A34R were used to set the gate for tetramer + cells. All pB7.2 tetramers identified distinct populations of positive cells (Fig. 2). To determine the kinetics of the epitope specific CD8^+^ T cell response, mice were infected with HMPV and euthanized on selected days post-infection. Lung and spleen lymphocytes were enumerated by tetramer staining (Fig. 3). Epitope-specific CD8^+^ T cells were more frequent in the lung compared to the spleen and peaked in the lung on day 13, though not all tetramer-specific cells peaked on the same day (Fig. 3). Four of these epitopes, M195, F97, N307, and N198 were validated in separate experiments using flow cytometry with doubly labelled pB7 tetramers compared to an irrelevant respiratory syncytial virus peptide NS-2_19_ (Supp. Figure 1). These data indicate that HLA-B7-restricted epitopes were recognized by CD8^+^ T cells in infected animals.

### BMDC vaccination with HMPV peptide reduces viral titers in transgenic HLA-B*07:02 mice

**Figure 4:**
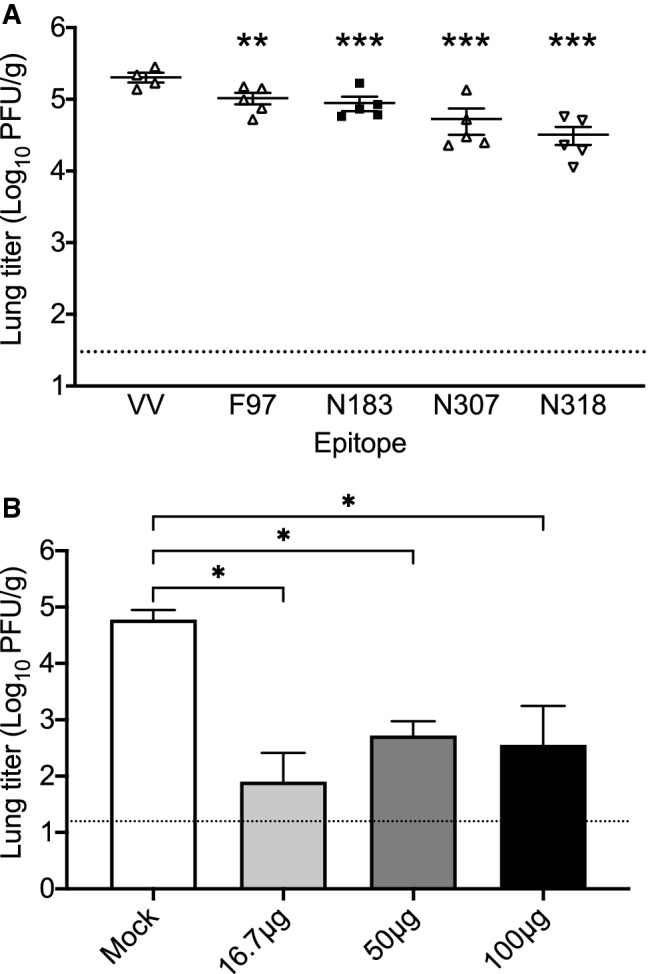
Epitope vaccination with HMPV peptide reduces lung virus titer in B7tg mice. (**A**) B7tg mice were vaccinated with LPS-matured bone marrow-derived DCs loaded with vaccinia virus (VV) or individual HMPV peptides, allowed to rest for 2 weeks, and then infected i.n. with 1 × 10^6^ PFU HMPV. (**B**) B7tg mice were vaccinated with combined HMPV peptides plus LPS, allowed to rest for 4 weeks, and then infected i.n. with 1 × 10^6^ PFU HMPV. In both experiments, lungs were collected on day 5 post-infection and HMPV titer determined using plaque assay. Error bars represent SEM. *P < 0.05, **P < 0.01, ***P < 0.001 compared to mock, one-way Anova with Dunnet’s correction for multiple comparisons. Dotted line indicates limit of detection.

To test the function of virus-specific CD8^+^ T cells, we used peptide-loaded, LPS-matured bone marrow-derived DCs to prime HMPV-specific CD8^+^ T cells^35,36^. We vaccinated B7tg mice subcutaneously with individual HMPV peptides or vaccinia virus A34R peptide. All mice were challenged with HMPV at 2 weeks post-immunization, and lungs harvested at the peak of viral replication (day 5). Mice vaccinated with the HMPV peptides had a modest reduction in viral titers compared to control mice vaccinated with the vaccinia-derived peptide (Fig. 4A). We next asked whether vaccination with a combination of epitope peptides combined with LPS adjuvant would provide more robust protection against challenge infection. Peptides N183, N198, N307, N318, M195, and F97 were administered to mice in three different dose schedules of 16.7 µg of each peptide + 10 µg LPS, 50 µg of each peptide + 10 µg LPS, or 100 µg each peptide + 10 µg LPS. All mice were challenged with HMPV at 4 weeks post-peptide immunization, and lungs harvested on day 5 post-infection. Each dosing regimen led to a significant reduction in lung virus titer on day 5 compared to mock, though there was no significant difference between the three dosing regimens (Fig. 4B). These data indicate that that vaccination with HMPV MHCI-restricted epitope peptides elicited immunity against HMPV that acted to reduce viral titers in vivo.

### Human HLA-B*07:02 PBMCs recognize HMPV peptides

**Figure 5:**
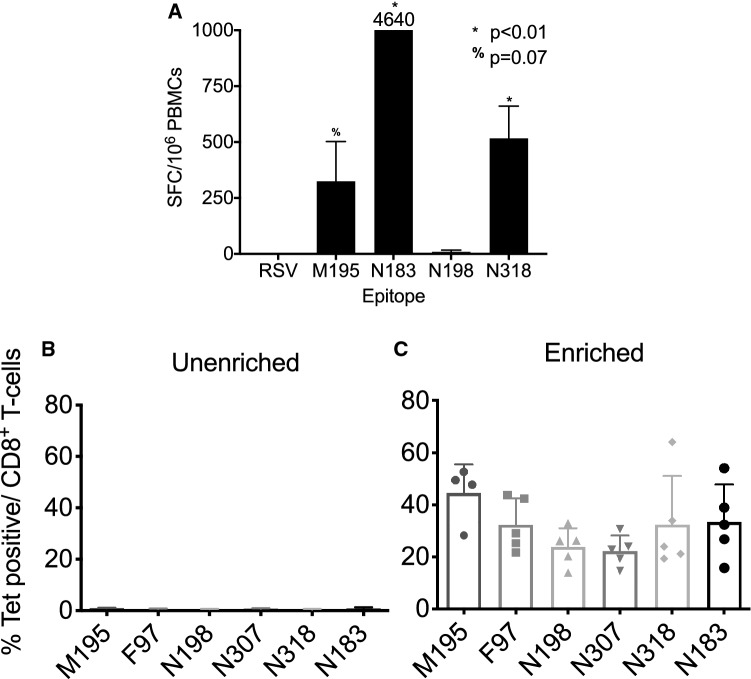
Human HLA-B*07:02 PBMCs recognize HMPV peptides. (**A**) Human HLA-B*07:02 PBMCs from a single donor were incubated with 10 μM HMPV peptides and recombinant human IL-2 for 7 days, IFNγ ELISPOT performed as described, and SFC expressed as average SFC/10^6^ human PBMC in triplicate wells corrected by subtracting SFC from irrelevant peptide control wells. (**B**) Human HLA-B*07:02 PBMCs from 5 distinct donors were incubated with APC-labeled HMPV tetramers, enriched through a magnetic column with anti-APC beads and both depleted flowthrough unenriched (left) and enriched (right) fractions analyzed via flow cytometry.

To test whether protective HLA-B7 epitopes identified in mice are shared by humans, we first performed ex vivo restimulation of PBMC from a single HLA-B7 donor. PBMC were isolated and cultured for 7 days with peptides M195, N183, N198, or N318 and recombinant IL-2. ELISPOT assay was used to determine reactivity to HMPV peptides or a control RSV peptide. The donor exhibited strong responses to epitopes M195, N183, and N318 (Fig. 5A). We then tested HLA-typed PBMCs from 5 individual de-identified donors each expressing at least one HLA-B*07:02 allele. Since we and others observed that the frequency of HMPV-specific CTLs is low in the peripheral blood of human donors^25–27^, we enriched the cells using a magnetic APC positive selection affinity column. PBMCs were passed through the column and both the enriched and depleted fractions were collected (Fig. 5B). HMPV-specific CD8^+^ T cells were detected in all donor samples, though the response varied between donors (Fig. 5B). Since all humans are infected with HMPV by 5 years of age^1,11,12^, and reinfections are common^3,4,9,14^, these data indicate that prior HMPV infection in humans elicits CD8^+^ T cells that recognize the HLA-B*07:02-trestricted epitopes identified in B7tg mice.

## Discussion

HMPV is a leading cause of human respiratory disease. An in-depth understanding of the human CD8^+^ T cell response will both inform studies of the HMPV immunity in humans and guide vaccine development. Neutralizing antibodies against the HMPV fusion (F) protein protect against replication and disease in animal models^37–39^. However, CD8^+^ T cells are required to clear HMPV infection in mice^16^; moreover, humans with impaired T cell immunity experience severe HMPV infection^8,9,17,27^, emphasizing the need for an effective vaccine that elicits both antibody and CD8^+^ T cell responses.

We used a combination of peptide screening approaches with in vivo experiments to identify HLA-B7-restricted epitopes that are recognized during HMPV infection. We discovered six new epitopes, including M195 as the immunodominant epitope in B7tg mice. All epitopes were highly conserved among HMPV strains of the four genetic lineages (not shown), suggesting the possibility of cross-protective cell-mediated immunity against HMPV as shown for poxviruses in the HLA-B7tg mouse^40^. Most were in matrix and nucleocapsid structural proteins, while the F97 epitope spans the conserved cleavage site of the F protein. The apparent immunodominance varies between the ELISpot and flow cytometry tetramer analyses (Figs. 1, 2, 3); this may reflect differences between peptide presentation by antigen-presenting cells in vitro and in vivo. Similar studies using the same HLA-B*0702 mice infected with influenza found that only 3–6 viral peptides were presented^41^, while epitope discovery of HLA-A*0201-restricted peptides in West Nile virus only found six peptides^42^. We previously used HLA-A*0201 transgenic mice to perform similar HMPV epitope mapping studies and identified six epitopes^25^. Mass spectrometry analysis of RSV-infected cells identified only nine naturally processed HLA-B27 peptides^43^. Thus, for viruses such as these with relatively small genomes, it may be that a limited number of peptides from the viral proteome can be presented by a single HLA molecule.

Of clinical/translational relevance, we tested the epitopes against PBMCs from HLA-B7 positive adults and demonstrated that the mouse epitopes were recognized by human CD8^+^ T cells as well. Since all humans are infected by HMPV in early life^1,11^, this presumably represents memory responses. The process of enrichment makes it challenging to assign dominance or determine precursor frequency. Due to the small number of cells, we did not perform in vitro functional assays (e.g., intracellular cytokine staining) to confirm that these were functional CD8^+^ T cells_._ Two of these epitopes, M195 and N307, were previously described in human PBMCs tested by ELISPOT^26^. Another study reported reactivity to peptide mixes from HMPV proteins in human PBMCs but did not map specific epitopes^27^. Due to the variability in peptide recognition between individuals, more extensive testing of human anti-HMPV CD8^+^ T cell responses against multiple donors would be needed to define the full HLA-B7 restricted repertoire in a human population.

Immunization of mice with individual peptides using DC vaccination demonstrated a modest reduction in viral titer, while combined vaccination with all 6 epitopes plus LPS adjuvant exhibited significant antiviral function in a vaccine-challenge model, with approximately 100-fold reduction in lung viral titer. Notably, all three doses reduced viral titers equally; this may reflect a threshold effect for CD8^+^ T cell induction^44^. These experiments do not determine the optimum dose, preparation, or route of epitope vaccination; however, not all CD8^+^ T cell epitopes are protective, even if immunodominant^45^.

Members of the HLA-B7 supertype collectively cover 43–57% of the human population across various ethnic groups and thus these epitopes are of broad clinical relevance^46^. However, similar work testing human PBMC responses to specific HLA-restricted viral peptides often finds a heterogenous response, varying from strong to absent^27,47^. Live challenge and live attenuated vaccine studies of HMPV have been conducted in human subjects, facilitating studies of T cell immunity to HMPV in humans^48,49^. The identification of HLA-B7-restricted HMPV epitopes recognized by human CD8^+^ T cells will facilitate further studies on the human response to HMPV infection and vaccines.

## Supplementary Information

Supplementary Information.
