# Supplementary material for: Novel HLA-B7-restricted human metapneumovirus epitopes enhance viral clearance in mice and are recognized by human CD8+ T cells
Source: Sci Rep. 2021 Oct 21;11:20769. doi: 10.1038/s41598-021-00023-0 (PMC8531189; doi:10.1038/s41598-021-00023-0)
Supplement: Supplementary file 1 — Supplementary Information. [file 41598_2021_23_MOESM1_ESM.pdf]

Supplemental Table 1

Individual HMPV and RSV peptides tested by ELISPOT. Peptides with spots above background in bold font.

ELISPOT #	Peptide	Peptide seq
1	MPV-L-1253	LPRFMSVNF
2	MPV-L-313	RLRVHGTVL
3	MPV-L-1253	LPRFMSVNFL
**4**	**MPV-F-97**	**NPRQSRFVL**
5	MPV-N-264	IARSSNNIM
**6**	**MPV-M-195**	**APYAGLIMI**
**7**	**MPV-M-195**	**APYAGLIMIM**
**8**	**MPV-N-307**	**SPKAGLLSL**
9	MPV-M2-1-150	LPREKLKKL
10	MPV-N-339	RGRVPNTEL
11	MPV-L-1198	VPVYNRQIL
12	MPV-L-1114	IPTKSNGRL
13	MPV-L-733	KTRCQMTSL
14	MPV-L-1270	RPMEFPASV
15	MPV-M2-2-5	MPCKTVKAL
16	MPV-N-151	RPSAPDTPI
17	MPV-M-160	IPAFIKSVSI
18	MPV-F-63	GPSLIKTEL
19	MPV-L-753	KPVKLSEGL
20	MPV-F-440	RPVSSSFDPV
21	MPV-L-1625	FPKITFERL
**22**	**MPV-N-198**	**YPRMDIPKI**
23	MPV-G-169	RTVRRTTTL
24	MPV-M-208	NPKGIFKKL
25	MPV-L-1607	SPMVNLTQV
26	MPV-M-12	IPYTAAVQV
**27**	**MPV-N-183**	**TVRRANRVL**
28	MPV-P-199	GPTAARDGI
29	MPV-P-29	KPSHKRSQSI
30	RSV-L-558	MPSHIQNYI
31	RSV-NS2-19	RPLSLETII
32	MPV-F-234	MPTSAGQIKL
33	MPV-F-451	FPEDQFNVAL
34	MPV-F-519	KPTGAPPEL
35	MPV-G-139	ASRTKTSPAV
36	MPV-G-162	SPPWAMTRTV
37	MPV-G-206	SPVSPQTSA
38	MPV-M-121	KPYGMVSKFV
39	MPV-M2-1-123	HVALHNLVL
40	MPV-M2-1-139	TPASLINNL

41	MPV-M2-1-147	LKRLPREKL
42	MPV-M-28	LPASLTIWF
43	MPV-M-43	TPPAVLLDQL
44	MPV-N-102	KIKNNKGEDL
45	MPV-N-151	RPSAPDTPII
46	MPV-N-154	APDTPIILL
47	MPV-N-183	TVRRANRVL
48	MPV-N-203	IPKIARSFY
49	MPV-N-234	SSTGSKAESL
50	MPV-N-296	GPESGLLHL
**51**	**MPV-N-318**	**CPNFASVVL**
52	MPV-N-92	VVLTRTYSL
53	MPV-P-29	KPSHKRSQSI
54	MPV-P-55	ISRPAKPTIL
55	MPV-P-60	KPTILSEPKL
56	MPV-SH-152	KPAVEVHHIV
57	MPV-SH-22	TIKDHSGKVL
58	MPV-L-139	IPSWVSNWF
59	MPV-L-290	STRFRNTLL
60	MPV-L-326	YPMYEVVLKL
61	MPV-L-366	HPMVDERDAM
62	MPV-L-415	WPKIKNLKVL
63	MPV-L-462	VPEKTNLEM
64	MPV-L-478	SPPKRLIWSV
65	MPV-L-488	YPKNYLPETI
66	MPV-L-513	KTRRVLEYYL
67	MPV-L-1040	TPVYPHGLRV
68	MPV-L-1054	LPFHKAEKVV
69	MPV-L-1372	SPDKIDMLTL
70	MPV-L-1417	ALACHWCGIL
71	MPV-L-1587	LTRLIRKKL
72	MPV-L-1603	TPIPSPMVNL
73	MPV-L-1607	SPMVNLTQVI
74	MPV-L-1647	LTRNYMILL
75	MPV-L-1728	YPLEYQRVI
76	MPV-L-1765	IHRVSKDAL
77	MPV-L-1803	RICTTYGTDL
78	MPV-L-52	SPVIEHVRL
79	MPV-L-649	AICADVADEL
80	MPV-L-814	HPTPIKKIL

Supplemental Figure 1

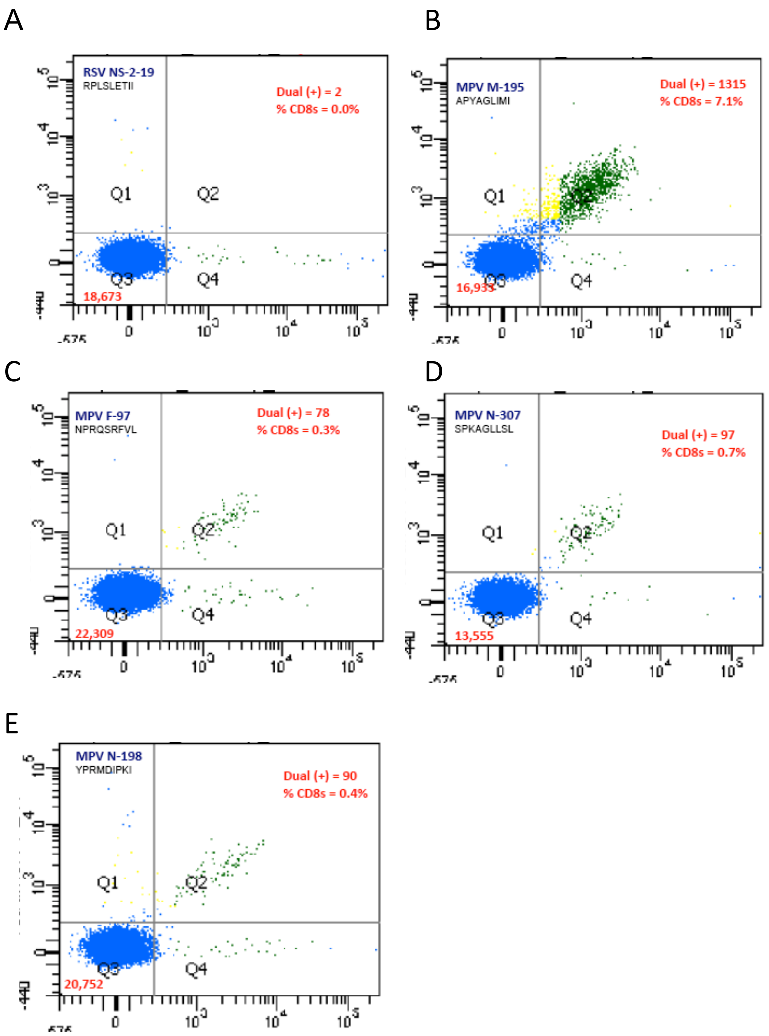

Supplemental Figure 1. Dual-color tetramer staining confirms specificity of HMPV B7 epitopes. B7tg mice were infected i.n. with $1x10^6$ PFU HMPV and spleens collected on day 10 post-infection. Live/dead dye and fluorescently labeled CD8 and CD3 antibodies were used to identify live $CD8^+$ T lymphocytes and both APC- and PE-labeled HMPV-specific tetramers, as well as an irrelevant respiratory syncytial virus (NS-2-19) epitope-loaded tetramer, were used to identify epitope-specific T_{CD8}.
